# Evaluation of Bacterial Cellulose/Alginate-Based Hydrogel and Frog Skin Dressings in Equine Skin Wound Healing

**DOI:** 10.3390/gels11020107

**Published:** 2025-02-03

**Authors:** Rita C. Campebell, Andressa B. Oliveira, Jéssyca L. A. Fagundes, Beatriz N. A. Fortes, Henrique C. Veado, Isabel L. Macedo, Bruno S. L. Dallago, Hernane S. Barud, José Adorno, Pablo A. V. Salvador, Paulo S. Santos, Márcio B. Castro

**Affiliations:** 1 Hospital Escola de Grandes Animais, Faculdade de Agronomia e Medicina Veterinária (FAV), Universidade de Brasília (UnB), Área Especial SRB, Galpão 4, Granja do Torto 70636-200, DF, Brazil; andressabo56@gmail.com (A.B.O.); jessycalauarf@gmail.com (J.L.A.F.); beatrizdna@gmail.com (B.N.A.F.); henriquemedvt@gmail.com (H.C.V.); isabeluanamacedo@gmail.com (I.L.M.); dallago@unb.br (B.S.L.D.); mbcastro@unb.br (M.B.C.); 2 Laboratório de Biopolímeros e Biomateriais (BIOPOLMAT), Departamento de Química, Universidade de Araraquara (UNIARA), Araraquara 14800-000, SP, Brazil; hsbarud@uniara.edu.br; 3 Asa Norte Regional Hospital, HRAN, SMHN Q2, Asa Norte, Brasília 70710-100, DF, Brazil; adornog@gmail.com; 4 Radiation Technology Center, CETER-IPEN-CNEN/SP, Sao Paulo 05508-000, SP, Brazil; ctr01@ipen.br (P.A.V.S.); psantos@ipen.br (P.S.S.)

**Keywords:** hydrogel, bacterial cellulose, frog skin, tissue regeneration, wound healing, horses

## Abstract

This study evaluates the wound-healing process in horses following the application of two treatment modalities: bacterial cellulose hydrogel with alginate (BCAW) and frog skin (FSW) dressings on experimentally induced skin wounds. Throughout the experiment, no clinical abnormalities were noted in the horses, although initial wound assessments indicated edema and sensitivity. Local hemorrhage was observed in some cases on Day 0, with granulation tissue formation evident by Day 14. Epithelialization began around Day 14 but did not reach complete healing in any group by Day 28. The analysis showed no significant differences in skin wound area or wound contraction rates among the treatment groups compared to control wounds (CWs) over the evaluation periods. Histopathological evaluations also indicated no significant differences in inflammatory responses or healing markers, such as fibroblast proliferation and neovascularization in skin wounds across groups. Despite expectations based on prior research in other species, the treatments with BCAW and FSW did not demonstrate substantial pro-healing effects in horses with induced skin wounds. These findings underscore the complexity of equine wound healing and suggest further investigation is needed to optimize treatment strategies in this species and enhance the translational potential for human clinical applications.

## 1. Introduction

Skin wounds are a frequent issue in equine practice due to horses’ instinctive over-responsiveness to external stimuli, often resulting in traumatic injuries. Complications during the wound-healing process in horses are common, leading to prolonged treatments, excessive scarring, and wound contamination [1]. The similarities between normal wound healing in humans and horses and the natural development of chronic wounds in both species make horses a valuable model for studying wound repair mechanisms. Additionally, horses are ideal models for testing novel therapies, with research potentially benefiting both veterinary and human medicine [2]. The healing process of large skin wounds involves a complex series of events that lead to complete tissue repair and re-epithelization. Generally, the management of skin wounds focuses on reducing local pain, hemorrhage, and infection to create favorable conditions for complete tissue regeneration [3,4,5].

In recent years, the development of biomaterials such as biopolymers has gained prominence in medicine. Among natural polymers, polysaccharides like cellulose and alginate have been explored for wound healing due to their availability and biocompatibility [6]. Bacterial cellulose (BC), or bio-cellulose, and its derivatives have shown promise in wound and burn healing thanks to their biological, physicochemical, and nanotechnological properties [7,8,9,10].

BC is synthesized by specific bacterial strains, most notably the Gram-negative *Gluconacetobacter xylinum*. This bacterium, naturally found in the environment, is the primary genus utilized for BC production in most studies due to its superior yield and quality compared to other strains. BC produced by *G. xylinum* exhibits exceptionally high purity, with a microfiber structure closely resembling that of algal and plant cellulose [11].

The biocompatibility of BC has been extensively studied, including a 120-day evaluation in rats with dural defects. The results demonstrated excellent mechanical stability, favorable biocompatibility, no immune response or chronic inflammation, and no evidence of neurotoxicity [12]. BC gel applied to equine lumbar wounds increased local perfusion and tissue metabolism, particularly within the first 14 days of healing [9].

Alginate hydrogels are widely utilized for their absorbent properties in drug and protein release, film and microsphere formation, and wound debridement [7]. When ionically incorporated into bacterial cellulose (BC) cross-linked hydrogels, alginate mimics the extracellular matrix and helps maintain moisture at the wound site. The ion exchange between calcium in the biomaterial and sodium in the wound environment forms a stable gel [13]. BC has shown a high capacity for treating wounds and burns, providing a moist environment. Alginate-based dressings offer several advantages, including effective exudate absorption, odor and pain reduction at the wound site, and the conversion of absorbed exudates into a gel through the ion exchange between calcium alginate and the wound. Additionally, alginates provide moisture, creating a protective barrier that inhibits bacterial invasion, reduces the risk of infection, and accelerates tissue regeneration [14,15]. Calcium alginate combined with BC has been shown to form hydrogels promoting wound debridement and efficient wound healing [6].

Biological dressings are categorized as either temporary or permanent. Permanent dressings integrate into the tissue, promoting and facilitating repair, while temporary dressings are removed once they have completed their role in stimulating tissue repair [16]. The use of biological dressings dates back to ancient times, with frog skin being cited as a treatment for wounds in the Ebers Papyrus circa 1500 BC [17]. The effectiveness of frog skin as a dressing has been linked to its lipid, protein, and peptide content and bioactive components in its secretions [18,19,20].

Frog skin has been successfully used in treating partial-thickness burns in human patients, primarily due to magainin, a dipeptide antibiotic with healing properties [21]. When used as a non-occlusive dressing in rabbits, studies have shown that frog skin enhances the healing process and demonstrates antimicrobial activity [22]. In a study of 16 human burn victims, frog skin proved to be an effective biological dressing with comparable results to Vaseline gauze and additional advantages such as ease of transport, storage, and antimicrobial properties [23].

In another study, frog skin powder was formulated into an ointment and tested on full-thickness wounds in mice, showing positive effects in reducing inflammation; enhancing fibroblast proliferation, collagen production, and neovascularization; and promoting faster wound closure with a reduced microbial load [24]. Furthermore, a novel amphibian skin peptide, OA-RD17, demonstrated promising therapeutic effects on wound healing in full-thickness and deep second-degree burns in mice and in ex vivo skin wounds of diabetic patients. It modulated cellular and molecular mechanisms such as macrophage proliferation, migration, and polarization (MAPK, NF-κB) and keratinocyte proliferation and migration (TLR4/MAPK/miR-632/Wnt/β-catenin molecular axis) [25].

This study aims to evaluate the effect of bacterial cellulose hydrogel with alginate and frog skin dressings in the wound healing of experimentally induced skin wounds in the lumbar region of horses.

## 2. Results and Discussion

### 2.1. Results

#### 2.1.1. Physical Assessment of Horses

All horses showed no clinical abnormalities during the physical assessments of the surgical wounds throughout the experiment. From D1 to D7, the wounds exhibited edema and sensitivity, with the horses showing increased reactivity during dressing applications.

#### 2.1.2. The Assessment of the Wounds

Local hemorrhage was observed on D0 in one animal at the CW and in two horses at the BCAW. Clots, edema, and hyperemia were present in all animals across all groups until D3. Crust formation was noted in most animals from D14 onward. Between D3 and D14, granulation tissue began to fill the wounds, appearing initially minimal on D3, irregular on D7, and fully developed across the wound bed by D14 in all groups.

**Figure 1:**
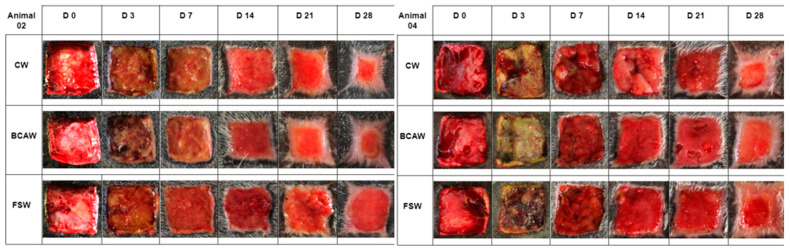
Photographic evaluation of wounds in the lumbar region of horses 02 and 04, from the control wounds (CWs), bacterial cellulose and alginate hydrogel wounds (BCAWs), and frog skin wounds (FSWs) on Days 0 (D0), 3 (D3), 7 (D7), 14 (D14), 21 (D21) and 28 (D28).

Epithelialization margins were noted in animals 01, 02, and 04 from D14 onward in all wound management treatments, while horses 03 and 05 displayed epithelialization from D21 onward, although complete healing was not achieved in any group. Epithelialization margins were clearly visible in different wound treatment protocols (Figure 1).

#### 2.1.3. Wound Area and Wound Contraction Rate

**Figure 2:**
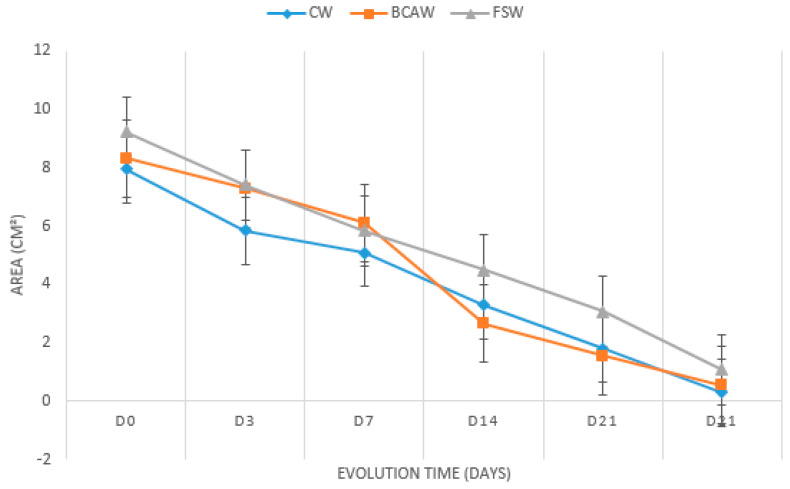
Mean wound areas in cm^2^ of the control wounds (CWs), wounds treated with bacterial cellulose hydrogel with alginate (BCAWs), and frog skin wounds (FSWs) in the lumbar region of horses on Days 0 (D0), 3 (D3), 7 (D7), 14 (D14), 21 (D21), and 28 (D28).

No statistical differences were observed between treatments in the mean wound areas in all evaluated periods. However, differences were detected in the wound-healing progress in the groups during the evaluation days. A marked reduction in the wound areas was detected in all treatments from Day 0 to D28, especially in D14 and D21 in the BCAWs. Even if not significant, the gross evaluation of the wound areas seemed to show large wounds for FGWs compared to BCAWs and CWs at the end of the experiment (Table 1 and Figure 2).

**Figure 3:**
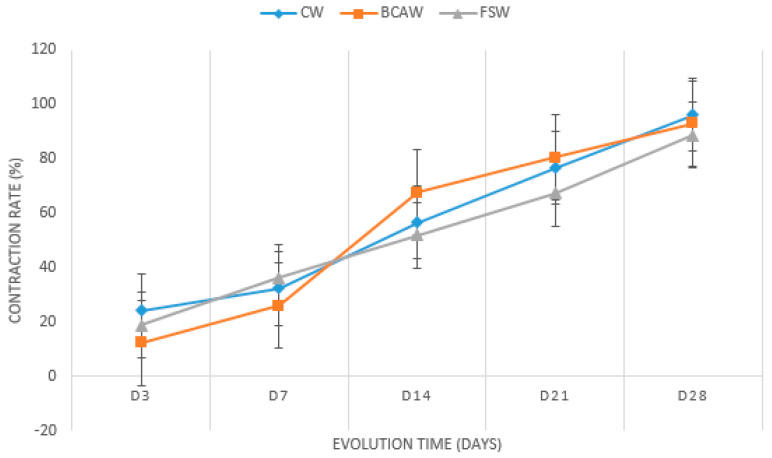
The mean wound contraction rate in percentages of the control wounds (CWs), wounds treated with bacterial cellulose hydrogel with alginate (BCAWs), and frog skin wounds (FSWs) in the lumbar region of horses on Days 0 (D0), 3 (D3), 7 (D7), 14 (D14), 21 (D21), and 28 (D28).

Similarly observed in the wound mean areas, no significant differences were detected in the wound contraction rates between CW, BCAW, and FGW during all time points. Additionally, the wound contraction rate grossly evaluated gave the impression of being lower in FGW than in BCAW and CW on Day 28. Some differences were detected in the evolution of wound contraction rates in the treatments throughout the evaluation periods. (Table 2 and Figure 3).

#### 2.1.4. Histopathological Evaluation

**Figure 4:**
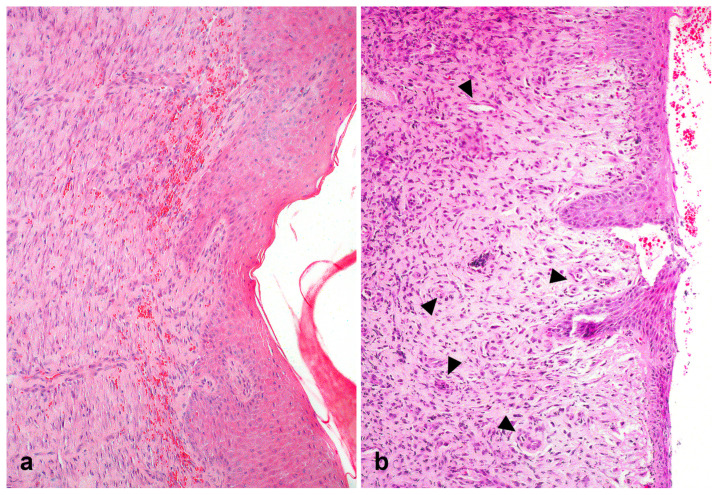
(**a**) Epithelialization, marked proliferation of fibroblasts and fibroplasia in a skin wound of a horse on Day 28 treated with bacterial cellulose hydrogel and alginate (BCAW) (H&E, objective 10×). (**b**) Prominent neovascularization (arrow heads) in a skin wound of a horse on Day 28 treated with frog skin (FSW) (H&E, objective 10×).

No statistical differences existed between the groups in the histopathological evaluation in any of the evaluated parameters. Additionally, there were no differences in fibroblast (Figure 4a) and neovascularization (Figure 4b) scores between treatment groups at all the evaluated time points.

### 2.2. Discusion

Horses share similarities with humans in the natural wound-healing process. In addition to both species developing different types of chronic wounds, horses serve as a relevant physiological model for studying wound repair mechanisms and evaluating potential therapies applicable in human clinical practice [2]. The skin architecture of humans and horses exhibits similarities, with both species having two primary layers that protect internal organs from mechanical damage and pathogen invasion. In humans, the epidermis comprises five layers of stratified epithelial cells, while in horses, it consists of stratified squamous cells. Both species have thick dermis layers, although humans have sparse hair follicles, while horses possess densely distributed follicles [26,27]. Studies on wound healing in horses, such as this one, may contribute as models for treating human skin lesions.

This study used sedation and local infiltrative anesthesia to ensure the animals’ comfort during surgical skin wound procedures, consistent with other experimental studies [28,29]. Additionally, non-steroidal anti-inflammatory drugs (NSAIDs) were administered during the first three days to effectively manage pain and edema, with only mild discomfort observed in the horses during dressing application in the initial post-surgical days. NSAIDs have similarly been employed in other studies on wound healing in horses [28,30,31], in which mild discomfort was also reported during dressing changes shortly after wound creation [32].

The design and size of the surgically-produced skin wounds, created with a template, were suitable for assessing treatments over an adequate period, as previously demonstrated [28,33]. The wound shape did not impact healing time in the horses [34], and using one side of the back for clinical evaluation and the opposite side for biopsy sampling prevented bias and interference in measuring wound area and contraction [29,30]. A clinical evaluation showed an initial inflammatory phase in all animals and groups following surgery, marked by hemorrhage, edema, hyperemia, and clot formation that persisted until D3 and was no longer evident by D7 [32].

In this study, exudate decreased as granulation tissue began filling the wounds but remained until closure. In CWs, exudate was noted until granulation tissue filled the wounds, after which they stayed dry. By D7, all animals had some granulation tissue, though not covering the entire wound area, similar to findings in other studies [28,35]. FSW exhibited three animals with prominent granulation between D14 and D21, and all groups on D28, but still with incomplete wound contraction.

Epithelialization of skin wounds began around D14 in most animals, becoming more pronounced in BCAWs on D21 and CWs on D28. Although other studies have shown a pro-healing effect, with good epithelial growth in frog skin-treated skin wounds in humans [23] and faster epithelialization in bacterial cellulose hydrogel-treated wounds in rats [36], this effect was not observed in our study. For both treatments (BCAW and FSW), no significant differences in wound area or contraction rate were observed compared to the control wounds by the end of the experiment (D28), consistent with findings in rat studies using bacterial cellulose hydrogel for experimental wounds [36]. The effect of cellulose gel with alginate on wound skin healing in horses is still lacking in the literature.

The pro-healing effects of cellulose gel combined with alginate are primarily attributed to its ability to retain moisture, creating an environment conducive to epithelialization by promoting epithelial cell migration into the moist wound area [36,37]. This property offers a significant advantage over bacterial cellulose alone or occlusive dressings, which generally maintain a low-humidity wound bed [38]. Studies on bacterial cellulose (BC) films incorporated with alginate, gelatin, and glycerol have shown that formulations made exclusively of BC and alginate exhibit excellent mechanical properties, such as high resistance and tensile strength, along with optimal hydrophilicity and a microenvironment favorable for keratinocyte and fibroblast growth [39]. Furthermore, bacterial cellulose/alginate hydrogels have demonstrated clinical efficacy; for example, in treating a diabetic ulcer, twice-weekly applications over 30 days led to an 84% reduction in wound size compared to the initial measurement [40].

Frog skin dressings have also shown promising healing properties, attributed to their porosity, which facilitates oxygen flow while preventing liquid or exudate buildup in rabbits [22] and humans [23]. Despite these demonstrated pro-healing effects of BC and alginate-based dressings and frog skin in other species, this study did not observe such effects in horses.

Despite minor variations, histopathological evaluations showed no significant differences across groups in terms of inflammatory response or wound healing markers, such as fibroblast presence and neovascularization scores. Although not statistically significant, BCAW demonstrated a slight reduction in neutrophil and histiocyte scores during the early and middle stages of healing. The cellulose/alginate gel has been shown to reduce neutrophil and macrophage activation, modulate the inflammatory phase, and enhance the healing of skin burns in rats [6]. It has also been associated with improved local perfusion and tissue metabolism in lumbar wounds in horses [9]. In rats, the topical application of frog skin lipid extract accelerated wound healing, likely due to its ability to shorten the inflammatory phase [41]. However, this effect was not observed in our study, similar to findings in dog integumentary wound healing [42]. Consequently, the wound-healing benefits of frog skin and bacterial cellulose hydrogel with alginate were not confirmed for experimentally induced skin wounds in horses.

## 3. Conclusions

Given the high prevalence of wounds and the associated healing challenges in horses, exploring various treatment modalities and protocols at different healing stages is essential to better understand morbidity and complication rates. This study demonstrated that surgically induced skin wounds in horses can serve as a reliable experimental model to evaluate the effects of topical products on cutaneous wound healing in this species. Although there are some similarities in the wound-healing process between horses and humans, the results observed in one species may not always be the same in another.

The use of bacterial cellulose hydrogel with alginate (BCAW) and frog skin (FSW) on experimentally induced skin wounds in horses did not demonstrate significant pro-healing effects when compared to control wounds. Similarly, no significant differences were observed between the use of BCAW and FSW on equine skin wound management, either grossly or histologically. Thus, despite the promising outcomes reported in other species, including humans and rodents, the expected anti-inflammatory and wound-healing benefits of BCAW and FSW were not observed in horses. These findings highlight the complexity of wound healing across species and suggest that further research is necessary to understand better the potential and limitations of these dressings in equine wound management, as well as to assess the relevance of this model in translating results to human clinical applications.

## 4. Materials and Methods

This manuscript was deposited as a preprint at DOI:10.20944/preprints202405.0098.v1.

### 4.1. Animals

Five healthy adult horses, consisting of four geldings and one mare, were selected for this study. Four were mixed breeds, and one was a Purebred Arabian, with a body weight average of 381.2 kg ± 57.07. The number of animals selected for this experiment is consistent with comparable studies in the field, which utilized six horses [29,33,43], five horses [32], and four horses [44,45]. This reflects a clear trend in equine wound research toward minimizing the number of animals involved, which aligns with ethical considerations and the principles of the 3Rs (Replacement, Reduction, and Refinement).

The horses, owned by the Large Animal Veterinary Hospital of the University of Brasília (UnB), had no history of skin diseases and underwent thorough clinical examinations and blood tests, including hematology and serum biochemistry for urea, creatinine, gamma-glutamyl transferase (GGT), aspartate aminotransferase (AST), and albumin. Throughout the experimental period, daily assessments were conducted, including heart rate (HR), respiratory rate (RR), mucous membrane color, capillary refill time (CRT), rectal temperature (T °C), intestinal motility, general demeanor, and appetite.

Prior to this study, the horses were dewormed using an oral paste containing ivermectin and praziquantel (Padock Plus NF^®^, CEVA, Paulínia/SP, Brazil). They received a prophylactic dose of tetanus antitoxin (Venco-sat^®^, Vencofarma, Londrina/PR, Brazil) via intramuscular injection. After the surgical procedures, the animals were housed in individual stalls under hygienic conditions and fed with commercial equine feed (Nutrina Equinos Premium^®^, Nutrina Rações, and Minerais, Brasília/DF, Brazil), coast-cross hay, and water ad libitum.

The study protocol was approved by the Ethics Committee on the Use of Animals (CEUA) at the University of Brasília (process No. 62/2019, approved on 21 August 2019), under the National Council of Ethics regulations. The horses used in this study had been abandoned in public areas and were collected by the Department of Agriculture of the Federal District (Brasília). Prior to the research, all horses received clinical care, vaccinations, deworming, and proper nutrition until they were deemed healthy and fit for participation. All horses were rehomed to new owners in compliance with animal welfare protocols after approximately 35 to 40 days of the experiment to allow complete wound healing.

### 4.2. Bacterial Cellulose Hydrogel with Alginate

**Figure 5:**
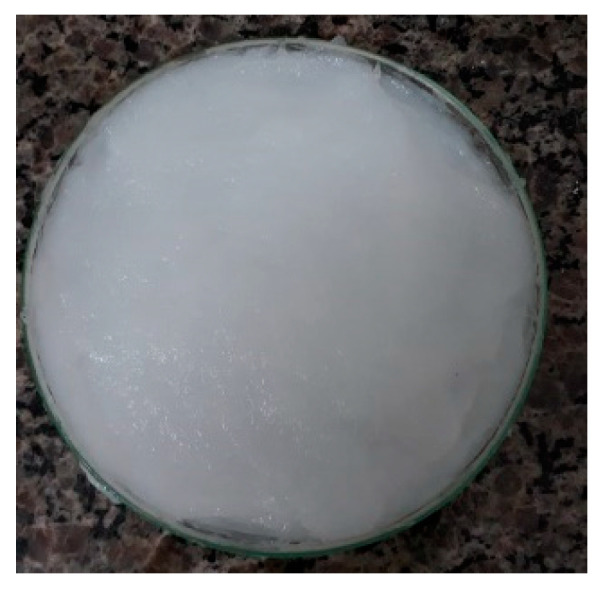
Cellulose-based hydrogel (1%) with alginate (Seven Indústria de Produtos Biotecnológicos LTDA).

The cellulose-based hydrogel (1%) with alginate was provided by Seven Indústria de Produtos Biotecnológicos LTDA (Figure 5). This product, currently in the development phase, is designed to aid in healing deep wounds and cavities in human medicine. This hydrogel was patented by the Brazilian company Seven Biotech (Londrina, Brazil) under the Brazilian Patent Office (INPI) in 2019—Process number: BR 10 2019 021848 7.

### 4.3. Frog Skin (Rana catesbeiana)

The frog skin (*Rana catesbeiana*) was supplied by Ranário Laranjeiras–Ranajax, located in Anápolis (GO, Brazil), a facility specializing in the cultivation of this anuran species to produce high-quality products and by-products in compliance with the “Code of Responsible Conduct for Frog Cultivation”, a document issued by the Secretariat of Aquaculture and Fisheries of the Presidency of the Republic (SEAP/PR) in 2002. The skin was sourced from healthy animals slaughtered at industrial processing plants inspected by the Federal Inspection Agency, which oversees the meat and by-products, including the skin.

**Figure 6:**
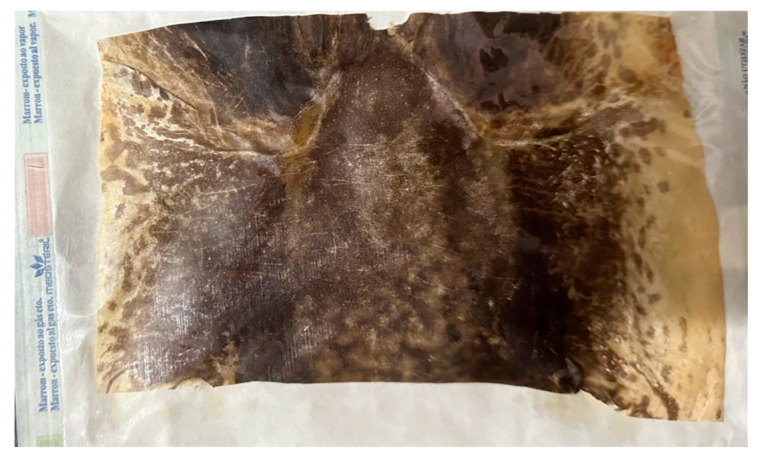
Frog skin packed in surgical-grade paper, sterilized by gamma ray.

Frogs weighing approximately 180 g were humanely euthanized at the frog farm, and the skin, including that of the body and extremities, was removed in a manner akin to peeling off a glove. The skin was then split longitudinally into 1 kg sub-lots, stored at 2 °C. The dehydration process was carried out using a dehydrator, in which a professional sanitized the metal plates with neutral soap. After drying, a layer of frog oil was applied to hydrate the skins. They were then exposed to a temperature of 28 °C for 45 min, allowing them to release from the plate and reach the desired condition. Once cooled, the skins were placed in surgical-grade packaging for sterilization by gamma radiation. This process was conducted using Co-60 gamma rays (with an energy of approximately 1.25 MeV) in a Gammacell GC220 MDS Nordion irradiator (Canada) at a dose rate of approximately 1.44 kGy/h, with a total dose of 25 kGy. The sterilization occurred at the Gamma Radiation Laboratory (GAMALAB), before being sent to INPE-USP in São Paulo/SP (Figure 6).

### 4.4. Experimental Induction of Skin Wounds

**Figure 7:**
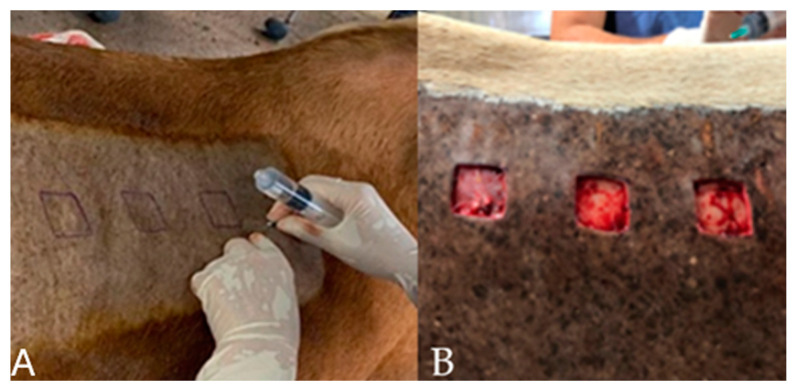
Experimental skin wounds on horses: (**A**) Mold marking; (**B**) Square-shaped wounds measuring 3.0 cm on each side, including skin and subcutaneous tissue.

Horses were sedated with 10% xylazine hydrochloride (Equisedan^®^, J.A. Saúde Animal, Patrocínio Paulista/SP, Brazil) at 1 mg/kg intravenous. A trichotomy was performed on both sides of the lumbar region, cranial to the tuber coxae and ventral to the transverse processes of the lumbar vertebrae from L1 to L6. Antisepsis was conducted using povidone-iodine scrub and 70% alcohol. Local infiltrative anesthesia was administered with 2% lidocaine without a vasoconstrictor (Lidocaine hydrochloride 2%^®^, Hipolabor Farmacêutica, Belo Horizonte/MG, Brazil) (Figure 7A).

On both sides of the lumbar region, cranial to the sacrum and ventral to the transverse processes of the lumbar vertebrae (from L1 to L6), three 3 cm^2^ skin flaps were excised from each side: one cranial, one medial, and one caudal, spaced 7.0 cm apart (Figure 7B). All incisions were performed using a sterilized 3.0 cm^2^ template to ensure consistent removal of the skin fragments. The depth of the incisions extended through the skin and subcutaneous tissue, exposing the muscle fascia. The wounds on the right side were designated for clinical and gross evaluation, while those on the left were reserved for biopsies and microscopic examination.

### 4.5. Treatments

Dressings were applied immediately after the surgical wounds were created and then once daily throughout the 28-day experimental period. All five horses received phenylbutazone at a dose of 4.4 mg/kg intravenously (Equipalazone^®^, CEVA, Paulínia/SP, Brazil) immediately after surgery and for the following three days to manage pain and edema.

**Figure 8:**
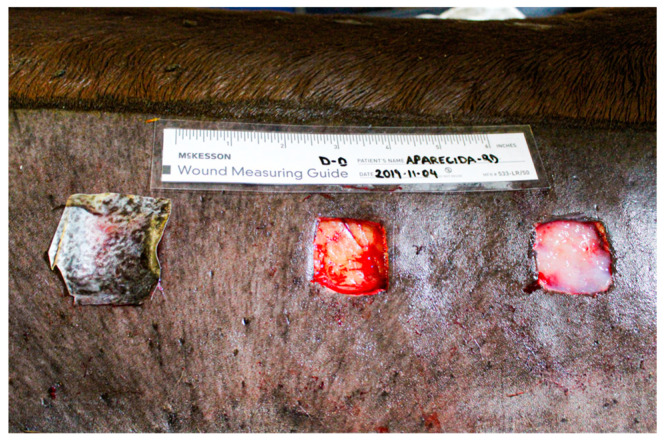
The treatment of skin wounds from the left to the right. Frog skin (FSW), control (CW), and bacterial cellulose and alginate hydrogel (BCAW) dressings are observed in the left lumbar region of a horse.

Before applying the dressings, all wounds were cleaned with lactated Ringer’s solution (Lactated Ringer Serum, Equiplex, Brazil). On both sides of the horse’s back, the first wound on each side was covered with frog skin (FSW) that had been rehydrated for 10 min in sterile lactated Ringer’s solution. The middle wounds (control wounds, CWs) were only treated with Ringer’s lactate solution and then bandaged with gauze. The third wound on each side received a layer of bacterial cellulose hydrogel ointment with alginate (BCAW), covering the entire wound area. The applied hydrogel seems to have a very low-density, almost paste-like structure (Figure 8).

### 4.6. Clinical and Photographic Evaluation of Wounds

**Figure 9:**
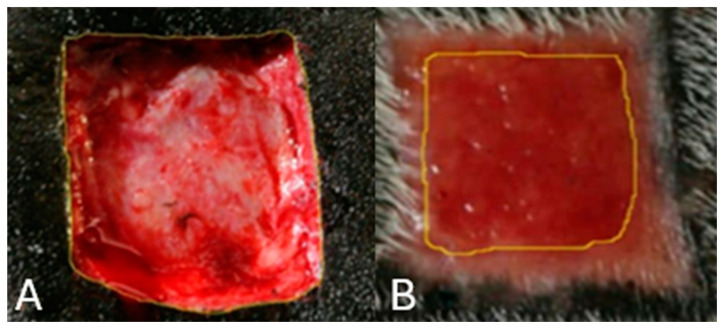
The assessment of control skin wounds in the lumbar region of a horse with the ImageJ software. (**A**) Wound area on D0. (**B**) Wound area on D14.

Clinical and photographic evaluations were performed on the day of surgery (D0) and Days 3 (D3), 7 (D7), 14 (D14), 21 (D21), and 28 (D28). The clinical evaluation assessed parameters such as edema, hyperemia, local hemorrhage, crust formation, clot presence, exudate type and quantity, granulation tissue development, and epithelialization. Photographic monitoring was used with a ruler to measure the wound area, allowing for an objective assessment of the wound contraction rates. Wound area measurements were processed using Image J2 Software (Wayne Rasband National Institutes of Health, Bethesda, MD, USA, http://rsb.info.nih.gov/ij/download.html, accessed on 15 August 2022) (Figure 9).

The degree of wound contraction was calculated based on the equation proposed by Ramsey et al. (1995) [46]: contraction rate (%) = 100 × (W0 − WA)/W0, where W0 represents the original wound area immediately after its creation, and WA represents the wound area at the evaluation time (D0, D3, D7, D14, D21, or D28). The result was expressed as a percentage.

Data on wound area and contraction rates were analyzed using a split-plot design with repeated measurements over time, followed by an analysis of variance (ANOVA) and Tukey’s test for mean comparisons. The data were assessed for normality and homogeneity of variances, and statistical significance was set at *p* < 0.05.

### 4.7. Biopsy and Histopathological Evaluation

Skin biopsies were performed using a 6 mm diameter punch under local infiltrative anesthesia with 1 mL of 2% lidocaine without vasoconstrictor (Lidocaine Hydrochloride 2%^®^, Hipolabor Farmacêutica, Belo Horizonte/MG, Brazil) on the day of surgery (D0) and on Days 3 (D3), 7 (D7), 14 (D14), 21 (D21), and 28 (D28). Biopsy samples were systematically collected from different areas of the wounds in a clockwise manner to avoid sampling the same site more than once. Tissue specimens were fixed in 10% buffered formalin (pH 7.0), routinely processed, embedded in paraffin, sectioned into 4 µm thick sections, stained with hematoxylin and eosin (H&E) staining, and evaluated under light microscopy.

The semiquantitative microscopic analysis of the wounds assessed inflammatory components, including neutrophils and histiocytes, as well as aspects of the healing process, such as fibroblast proliferation and neovascularization. Pathological changes were classified based on severity as (−) absent, (+) mild, (++) moderate, and (+++) marked. The median scores from the histopathological evaluation were analyzed using the Kruskal–Wallis test. All statistical analyses were performed using SAS^®^ software (version 9.4, Cary, NC, USA), with a significance level of 5%.

## Figures and Tables

**Table 1:** Mean and standard deviation of wound areas (cm^2^) of the control wounds (CWs), wounds treated with bacterial cellulose hydrogel with alginate (BCAWs), and frog skin wounds (FSWs) in the lumbar region of horses on Days 0 (D0), 3 (D3), 7 (D7), 14 (D14), 21 (D21), and 28 (D28).

Treatment	Wound Area (Mean—cm^2^)												
	D0		D3		D7		D14		D21		D28		Value *p*
	Mean	SD	Mean	SD	Mean	SD	Mean	SD	Mean	SD	Mean	SD	
CW	7.94A	2.55	5.84AB	1.22	5.07B	1.06	3.29BC	1.68	1.79CD	1.32	0.31D	0.30	<0.05
BCAW	8.32A	1.67	7.29A	1.55	6.11A	1.16	2.65B	0.99	1.54B	0.83	0.54B	0.31	
FSW	9.23A	1.37	7.40AB	1.46	5.85BC	2.28	4.50CD	2.08	3.08DE	1.60	1.09E	0.72	
Value *p*	ns		ns		ns		ns		ns		ns		

Different uppercase letters in the same row differ form each other using ANOVA. (ns: not significant).

**Table 2:** The mean and standard deviation of the contraction rate (%) of the control wounds (CWs), wounds treated with bacterial cellulose hydrogel with alginate (CBCAWs), and frog skin wounds (FSWs) in the lumbar region of horses on Days 0 (D0), 3 (D3), 7 (D7), 14 (D14), 21 (D21), and 28 (D28).

Treatment	Contraction Rate (%)										Value *p*
	D3		D7		D14		D21		D28		
	Mean	SD	Mean	SD	Mean	SD	Mean	SD	Mean	SD	
CW	24.40A	11.86	32.28AB	17.95	56.57BC	24.48	76.51CD	18.81	96.08D	3.92	<0.05
BCAW	12.24A	5.57	26.07A	7.52	67.68B	10.97	80.34B	12.33	92.87B	5.38	
FSW	18.83A	14.32	36.14AB	23.85	51.95BC	18.68	67.39CD	14.87	88.60D	6.53	

Different uppercase letters in the same row differ from each other using the ANOVA.

## Data Availability

The data presented in this study are available on request from the corresponding author.
